# Memory formation as selection: an eligibility-based architecture

**DOI:** 10.3389/fnsys.2026.1772781

**Published:** 2026-10-07

**Authors:** Athanasios Rizos

**Affiliations:** Independent Researcher, Thessaloniki, Greece

**Keywords:** eligibility-based architecture, memory formation, memory organization, mnemonic architecture, relational integration, selective stabilization

## 1 Introduction

Memory is often discussed in terms of encoding strength, retention, and retrieval success. Yet these dimensions do not fully explain how remembered information acquires structure within a larger mnemonic system. Strongly retained or highly accessible traces need not be broadly integrated, and their persistence or vividness alone does not establish that they will support abstraction, flexible transfer, or cross-context inference (Preston and Eichenbaum, 2013; Schlichting and Preston, 2015). What remains insufficiently specified, therefore, is not only why some information persists, but how persistent information becomes organized within the long-term architecture of memory.

Existing accounts have clarified important components of this problem. Work on attentional allocation, salience, precision weighting, and neuromodulatory gain has described how incoming signals may receive different processing and learning priority (Aston-Jones and Cohen, 2005; Friston, 2010; Feldman and Friston, 2010; Hasselmo, 2006). In parallel, relational memory theories have emphasized that memory depends not only on persistence, but on the binding of events, contexts, and higher-order regularities into structures that support flexible cognition (Eichenbaum, 2004; Preston and Eichenbaum, 2013; Eichenbaum and Cohen, 2014). These accounts address important aspects of selection, plasticity, and relational binding, but do not by themselves specify how differences in persistence and structural incorporation accumulate into different forms of long-term mnemonic organization.

The present article treats selection and mnemonic architecture as recursively coupled. The properties of incoming activity, the current state of the system, and its pre-existing organization jointly constrain which patterns become eligible for plastic modification. Selective stabilization concerns the degree to which eligible activity acquires persistence and recurrent availability, whereas relational integration concerns whether and how stabilized activity becomes incorporated into broader pre-existing representational structure. Through repeated learning episodes, stabilization and integration modify this structure and thereby alter the conditions of future selection.

Selective stabilization and relational integration are not proposed as alternative labels for episodic and semantic memory. Established memory categories describe forms or systems of memory, whereas the present constructs describe cross-cutting architectural dimensions that may operate within and across those categories (Renoult et al., 2019). The framework is concerned with whether selected information persists and how it contributes to broader mnemonic organization, rather than with replacing conventional classifications of memory.

The argument developed here is conceptual and deliberately non-exhaustive. The article does not propose a new synaptic learning rule, nor does it aim to replace established computational or neurobiological theories of memory. Instead, it offers a systems-level architecture for describing how eligibility, selective stabilization, and relational integration may interact across repeated learning episodes to produce enduring differences in mnemonic organization. Neurobiological mechanisms are treated as constraints and possible implementations of the processes described, not as one-to-one correlates of the proposed architectural dimensions. The goal is to clarify an explanatory level between transient processing priorities and the long-term structural organization of memory.

## 2 An eligibility-based architecture for memory formation

### 2.1 Memory formation as a selection process

Memory formation is often described as successful encoding followed by stabilization or consolidation over time. Such descriptions address how a trace may persist, but do not fully explain why only some ongoing patterns acquire access to enduring modification or how the current organization of the system biases that access. Research on memory allocation and synaptic tagging similarly indicates that activation may create an opportunity for lasting change without guaranteeing that such change will occur (Redondo and Morris, 2011; Josselyn and Frankland, 2018). The present framework uses selection to identify this transition from transient activity to potential structural consequence.

Selection is not treated as an independent gate acting on neutral input. It emerges from the interaction among the properties of incoming activity, the current state of the system, and its pre-existing representational organization. Intense or novel activity may acquire learning priority partly because of its input properties, while existing organization may preferentially recruit patterns through their relation to prior representations and established processing pathways (Aston-Jones and Cohen, 2005; Friston, 2010; Feldman and Friston, 2010). These influences are neither mutually exclusive nor fixed in their relative contribution. Selection is therefore neither purely stimulus-driven nor solely mnemonic, but dependent on the interaction between ongoing activity and the system in which it occurs.

Within this account, selection refers to the processes through which active patterns acquire eligibility, whereas eligibility refers to the resulting temporary, state-dependent susceptibility to plastic modification. An active pattern may be processed without becoming eligible, and an eligible pattern may still fail to achieve enduring stabilization. Eligibility should therefore not be equated with memory formation or persistence. It functions as a bridge between transient processing and the possibility of lasting structural change. Although this formulation is compatible with biological accounts of synaptic tagging and eligibility traces, eligibility is used here at a broader functional level and does not designate a specific synaptic mechanism or a new learning rule (Redondo and Morris, 2011; Gerstner et al., 2018).

The framework does not require selection to be exclusive to memory. Attentional selection and salience detection operate across cognitive domains and may bias which patterns become eligible for plastic modification. Their mnemonic consequences, however, depend on the current state and prior organization of the system. As mnemonic architecture develops, its established representational structure may increasingly constrain which patterns acquire learning priority, without making selection an exclusively mnemonic operation. The relevant question is therefore not whether memory possesses a unique selection mechanism, but how transient processing priorities become translated into persistent and differently organized mnemonic change.

Eligibility alone is not sufficient to determine the outcome of memory formation. Once a pattern has become eligible, two further questions arise: the degree to which it becomes selectively stabilized and recurrently available, and whether and how stabilized activity becomes incorporated into broader pre-existing representational structure. The following sections develop these dimensions as selective stabilization and relational integration.

### 2.2 Selective stabilization

Once an active pattern has acquired eligibility, it may or may not produce a persistent mnemonic consequence. Selective stabilization denotes the degree to which eligible activity becomes enduring and recurrently available, thereby retaining the capacity to influence subsequent processing. It does not refer to the upstream processes through which learning priority is assigned. The qualifier selective indicates that stabilization depends on prior eligibility and on the conditions under which eligible activity is maintained; it does not imply the operation of a second, independent selection mechanism.

Eligibility creates the possibility of plastic modification but does not guarantee the durability of its consequences. Some eligible patterns may remain weak or transient, whereas others may acquire sufficient stability to support later accessibility and recurrent influence on processing. Selective stabilization is therefore graded and conceptually separates susceptibility to plastic change from the persistence of the resulting modification.

Attentional allocation, salience-related processing, and precision weighting may contribute to determining which patterns become eligible, but they do not themselves constitute selective stabilization. Neuromodulatory systems should likewise not be confined to the transition from activity to eligibility, because they may constrain encoding and plastic modification across several stages. Cholinergic and noradrenergic influences are therefore treated as biologically plausible constraints on the proposed architecture, rather than as one-to-one correlates of selective stabilization or relational integration. Their mnemonic consequences depend on the current state and prior organization of the system in which they operate (Aston-Jones and Cohen, 2005; Hasselmo, 2006).

Selective stabilization addresses the persistence and recurrent availability of eligible activity, not the pattern or extent of its incorporation into broader representational structure. A strongly stabilized pattern may remain accessible and locally robust while being only weakly integrated with other representations. This further architectural dimension is addressed by relational integration.

### 2.3 Relational integration

Whereas selective stabilization concerns the persistence and recurrent availability of eligible activity, relational integration concerns its structural fate within the mnemonic system. It denotes whether, to what extent, and in what pattern stabilized activity becomes incorporated into broader pre-existing representational structure. Through such incorporation, stabilized patterns may become linked to prior episodes, contextual cues, conceptual relations, and higher-order regularities rather than remaining comparatively isolated. In this context, how refers to the pattern and scope of structural incorporation, not to a single underlying biological mechanism.

Relational integration is therefore not equivalent to encoding strength or retention. A pattern may be strongly stabilized, readily accessible, and behaviorally influential while remaining only weakly connected to wider representational structure. Measures of persistence, vividness, or retrieval accessibility cannot by themselves establish the degree of relational integration. Its distinctive contribution lies in allowing retained information to participate in broader organization and thereby support flexible retrieval, abstraction, transfer, and cross-context inference (Eichenbaum, 2004; Preston and Eichenbaum, 2013; Schlichting and Preston, 2015).

At a systems level, relational integration is compatible with accounts of hippocampal relational binding and interactions between the hippocampus, prefrontal systems, and distributed cortical representations (Preston and Eichenbaum, 2013; Eichenbaum and Cohen, 2014). These systems provide plausible biological constraints and possible implementations, but relational integration is not identified with a particular region or circuit. Nor is it another term for semantic memory. Episodic and semantic memory describe established forms or systems of memory, whereas relational integration describes a cross-cutting architectural dimension that may operate within and across them.

Across repeated learning episodes, differences in relational integration may accumulate into distinct tendencies of long-term mnemonic organization. Broad incorporation may contribute to distributed relational structure, whereas selective stabilization without corresponding integration may produce persistent but comparatively isolated representations. These outcomes are graded organizational tendencies rather than fixed memory types. Their cumulative expression at the level of the wider mnemonic system is considered in the following section.

### 2.4 Global memory organization

Long-term mnemonic organization emerges from the repeated interaction of eligibility with the architectural dimensions of selective stabilization and relational integration, rather than from any one component in isolation. Individual learning episodes may produce relatively local changes, but differences in which patterns become persistent and in how they are incorporated accumulate over time into large-scale representational structure. This relationship is recursive: the resulting architecture subsequently constrains which activity becomes eligible and how future information can be stabilized and incorporated.

At this level of description, global memory organization refers to the large-scale relational patterning of stabilized representations: the degree and manner in which they become interconnected, mutually constraining, and available for reconfiguration beyond the conditions of their original acquisition. This formulation is compatible with network accounts emphasizing the interplay between integration and segregation, while remaining focused on the organization of mnemonic representations rather than proposing a direct mapping onto a particular anatomical network (Bullmore and Sporns, 2009; Sporns, 2013).

When eligible patterns are sufficiently stabilized and broadly incorporated into pre-existing representational structure, repeated learning may bias the system toward distributed relational organization. In this organizational tendency, representations remain differentiated while participating in wider relational structure, allowing retained information to support abstraction, generalization, flexible retrieval, and transfer across contexts (Preston and Eichenbaum, 2013; Schlichting and Preston, 2015). This outcome does not require equality between selective stabilization and relational integration or a static equilibrium between them. It reflects their effective conjunction across repeated learning episodes.

By contrast, when selective stabilization repeatedly occurs without correspondingly broad relational integration, memory organization may become fragmented or weakly integrated. Under these conditions, representations may remain persistent, vivid, or readily accessible while being only narrowly connected to wider mnemonic structure. Such memories can retain substantial behavioral influence without supporting the same degree of abstraction, generalization, or cross-context inference (Eichenbaum, 2004; Schlichting and Preston, 2015). Fragmentation in this sense does not imply forgetting, weak encoding, or pathology. It denotes a graded organizational tendency in which locally robust representations contribute only to a limited extent to global relational structure.

Experiences with similar levels of retention may therefore have different architectural consequences. One stabilized pattern may become incorporated into a distributed relational system and participate in flexible recombination, whereas another may persist primarily as a locally strong but comparatively isolated representation. Over time, the accumulation of such differences modifies the architecture that constrains subsequent selection and learning. Global memory organization is thus both an outcome of prior stabilization and integration and a condition shaping their future operation.

### 2.5 Local structural features: local-core deepening

Global memory organization does not fully specify the internal structure of a mnemonic system. Architectures with different large-scale organizational tendencies may also contain representational clusters that vary in their local density, stability, and degree of specialization. The term local-core deepening refers to the progressive strengthening of internal relational organization within a circumscribed representational domain across repeated learning episodes. It describes a secondary structural feature of the emerging architecture, rather than an additional mechanism or independent architectural dimension.

Local-core deepening may arise when related patterns within a restricted domain repeatedly become eligible, are selectively stabilized, and are reactivated or incorporated in relation to one another. Over time, these recurrent dynamics can produce an internally dense cluster whose elements are strongly interconnected, even when the cluster remains only weakly connected to the wider mnemonic architecture. This process is also recursive: as local organization deepens, the existing cluster may increasingly constrain which related activity becomes eligible and how it is subsequently incorporated.

Strong local organization should therefore not be equated with broad relational integration. A memory system may contain highly elaborated local cores while remaining globally fragmented or weakly integrated when connections between these cores and the wider representational architecture are limited. Conversely, within a more distributed organization, local-core deepening may support specialization, domain sensitivity, and efficient access to highly practiced material while remaining connected to broader relational structure. Its functional significance depends not simply on local density, but on the relation between the local core and the architecture in which it is situated.

This distinction helps explain how memory may exhibit uneven profiles of robustness, accessibility, and domain-specific competence without requiring qualitatively different mnemonic mechanisms. Repeated engagement within a restricted domain may produce depth, speed, and internal coherence, but these properties do not by themselves entail flexible transfer, abstraction, or cross-context inference. Local-core deepening can therefore occur within different forms of global organization, allowing an architecture to be locally deep yet globally narrow, or globally distributed while containing selectively specialized domains (Figure 1).

**Figure 1:**
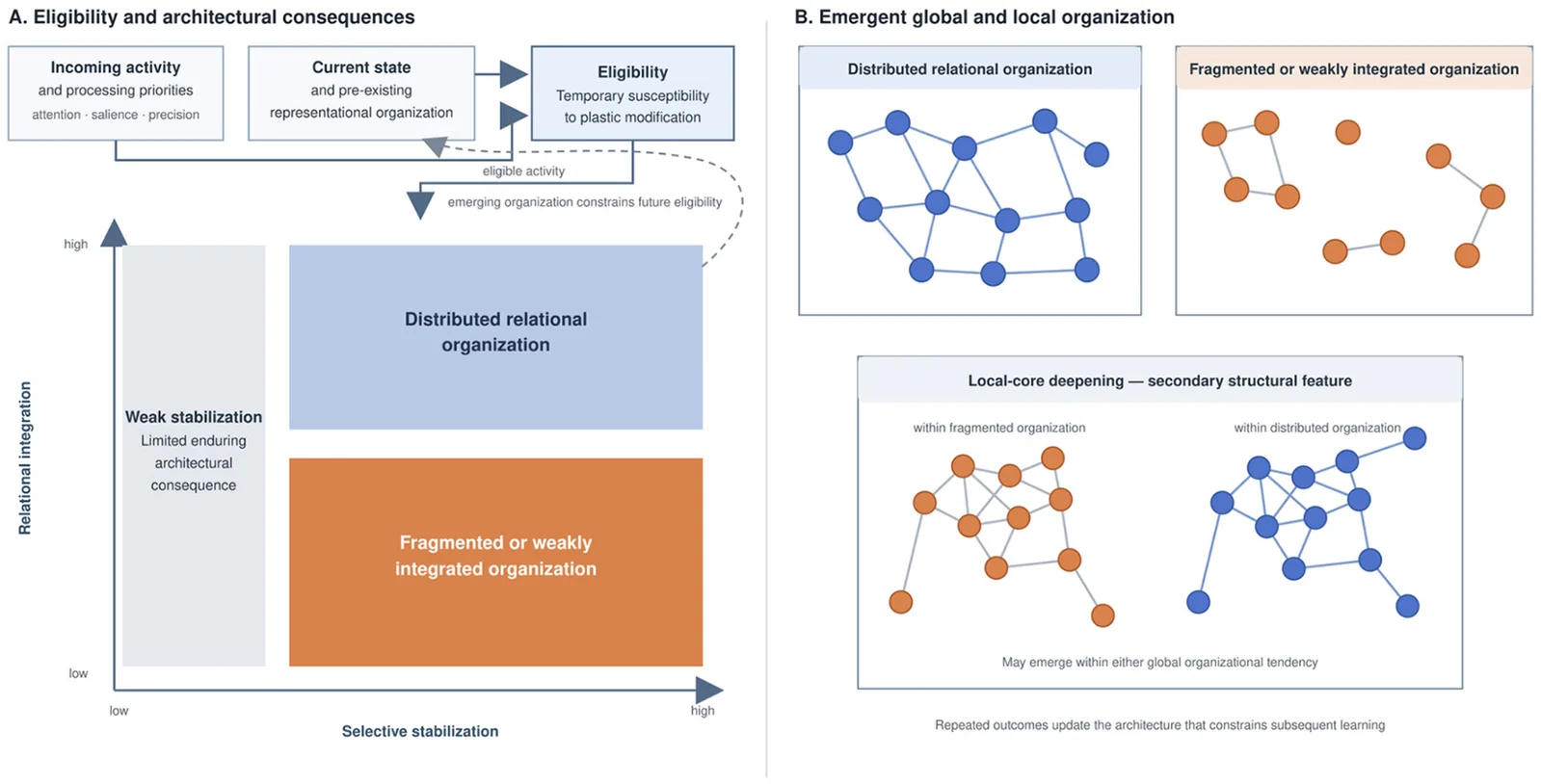
Eligibility-based architecture of memory formation. **(A)** The properties of incoming activity, transient processing priorities, the current state of the system, and its pre-existing representational organization jointly constrain which active patterns acquire eligibility for plastic modification. Attention, salience, and precision weighting may contribute to this process but do not themselves constitute selective stabilization. Among eligible patterns, selective stabilization refers to the degree to which activity acquires persistence and recurrent availability, whereas relational integration refers to whether, to what extent, and in what pattern stabilized activity becomes incorporated into wider representational structure. Activity that is only weakly stabilized has limited enduring architectural consequence. Strong selective stabilization accompanied by broad relational integration may contribute to distributed relational organization, whereas strong stabilization with limited integration may contribute to fragmented or weakly integrated organization. Neuromodulatory systems may constrain several stages of this process and are not represented as one-to-one counterparts of either architectural dimension. **(B)** The resulting organizational tendencies are illustrated as differences in large-scale mnemonic structure. Distributed relational organization is characterized by broader interconnection among differentiated representations, whereas fragmented or weakly integrated organization contains persistent but more sparsely interconnected representations. Local-core deepening is shown as a secondary structural feature that may emerge within either global tendency, reflecting increased internal density within a restricted representational domain without determining the organization of the system as a whole. Across repeated learning episodes, these outcomes modify the pre-existing architecture and thereby alter the conditions of subsequent eligibility, stabilization, and integration.

### 2.6 Relation to predictive processing

The present framework is compatible with predictive processing accounts but is not reducible to them. Within predictive processing, precision refers to the estimated reliability or confidence assigned to prediction errors, commonly formalized in relation to inverse variance, and regulates their influence on updating, attention, and learning (Friston, 2010; Feldman and Friston, 2010). Precision is therefore not synonymous with salience. Salience concerns the relevance or significance of activity to the system, whereas precision concerns its estimated reliability. The two may interact in determining learning priority, but they describe conceptually distinct properties.

Within the present framework, precision weighting is treated as one possible contributor to eligibility. By regulating the influence exerted by ongoing activity on updating, it may bias which patterns become temporarily susceptible to plastic modification. Precision weighting does not, however, constitute eligibility itself, nor does it define selective stabilization. Eligibility concerns susceptibility to modification, whereas selective stabilization concerns the degree to which eligible activity subsequently acquires persistence and recurrent availability. The term precision is therefore used here only in this technical and limited sense.

Predictive processing also addresses the formation and revision of hierarchical generative models. The present argument is not that such accounts cannot accommodate enduring mnemonic organization. Rather, precision weighting alone does not determine whether a particular update becomes persistent or how persistent change is incorporated into broader representational structure. The framework isolates these as distinct architectural questions through selective stabilization and relational integration. Consequently, activity exerting a strong influence on updating may contribute to a robust but comparatively local trace, or it may become incorporated into a distributed relational organization that supports abstraction, transfer, and cross-context inference (Preston and Eichenbaum, 2013; Schlichting and Preston, 2015).

The eligibility-based architecture should therefore be understood as operating at a complementary level of description. It does not replace predictive processing or propose an alternative computational rule. Its contribution is to specify how transient differences in learning priority may be translated into persistent and differently organized mnemonic change, and how the accumulation of such differences across repeated learning episodes may shape enduring mnemonic architecture.

## 3 Predictions and empirical implications

The framework generates empirically testable predictions from the distinction among learning priority, persistence, and structural incorporation. Its central empirical implication is that these properties should not be assumed to covary. Experimental designs should therefore distinguish indices of early selection or updating from measures of delayed retention and from outcomes that depend on relational organization, such as transfer, abstraction, and cross-context inference.

First, the framework predicts that comparable degrees of selective stabilization need not entail comparable relational integration. Experiences associated with comparable levels of delayed retention or retrieval accessibility may differ substantially in whether, to what extent, and in what pattern they become incorporated into wider mnemonic organization. Persistence, vividness, or rapid accessibility should therefore not be treated as sufficient indicators of relational integration (Preston and Eichenbaum, 2013; Schlichting and Preston, 2015). Equally memorable experiences need not be equally integrated.

Second, manipulations that increase salience, attentional priority, or precision-weighted influence should affect eligibility more directly than they determine long-term mnemonic organization. Increased learning priority may raise the probability that activity becomes susceptible to plastic modification, but it should not by itself specify whether that activity becomes durably stabilized or broadly incorporated. Material showing comparable early prioritization may therefore produce different long-term outcomes depending on subsequent stabilization and relational integration (Friston, 2010; Feldman and Friston, 2010).

Third, repeated eligibility and selective stabilization within a restricted representational domain should promote local-core deepening without necessarily increasing global relational integration. Recurrent engagement with related material may produce rapid access, internal coherence, and domain-specific robustness while leaving broader transfer and cross-domain organization comparatively unchanged. Once established, such local structure should also bias subsequent learning: new activity related to an existing core should be more readily recruited into that domain than comparably prioritized but unrelated activity. The expected signature is therefore asymmetric development of mnemonic organization—local structural deepening without corresponding expansion of global integration.

Fourth, conditions that promote relational incorporation should disproportionately improve outcomes that depend on flexible structural use rather than persistence alone. They should support transfer across contexts, relational generalization, and inferential use of prior knowledge even when delayed retention remains comparable to conditions that primarily enhance selective stabilization (Preston and Eichenbaum, 2013; Eichenbaum and Cohen, 2014). The most informative tests would therefore contrast retention-sensitive measures with integration-sensitive outcomes rather than relying on recall performance alone.

More generally, individual, developmental, and state-dependent variation may be better characterized through differences in the relation among eligibility, selective stabilization, and relational integration than through encoding strength alone. The decisive tests of the framework are those that can dissociate persistence from integration, local reinforcement from global organization, and accessibility from flexible relational use.

The framework would be weakened if measures of relational organization were consistently explained by retention strength alone, if increased learning priority reliably determined broad relational integration independently of subsequent mnemonic processes, or if established representational structure exerted no systematic influence on later learning. Support would instead come from evidence that comparable levels of retention can produce different integration-sensitive outcomes and that persistent traces vary in transfer, abstraction, and inferential utility as a function of their structural incorporation.

## 4 Scope and limitations

The present framework is intended as a systems-level account of how the consequences of repeated learning contribute to enduring mnemonic organization, rather than as a complete theory of learning, plasticity, or neural computation. It does not propose a new synaptic or computational learning rule. Instead, it identifies an explanatory level between transient processing priorities and enduring representational architecture. The account is therefore intended to complement, rather than replace, models of synaptic modification, hippocampal binding, systems consolidation, and predictive processing.

The proposed constructs should not be expected to map one-to-one onto particular neural regions, circuits, or neuromodulatory systems. A single biological mechanism may constrain more than one stage of the proposed architecture, and each architectural dimension may depend on multiple interacting mechanisms. Sleep, consolidation dynamics, motivational and affective states, task demands, developmental history, prior knowledge, and broader neurobiological conditions may influence eligibility, selective stabilization, relational integration, or their interaction. These influences operate across different timescales and are not necessarily external to the architecture: developmental history and prior knowledge, for example, may already be expressed in the system's current state and pre-existing representational organization. The present account does not attempt to model these contributions exhaustively.

A further limitation concerns empirical resolution. Eligibility, relational integration, and global memory organization are less directly observable than standard measures of encoding performance or retrieval success. The value of the framework therefore depends on whether future studies can operationally distinguish early learning priority from persistence, and persistence from structural incorporation. Designs combining retention measures with outcomes sensitive to transfer, abstraction, contextual flexibility, and inferential use will be needed to determine whether these properties dissociate in the manner proposed. Until such distinctions are demonstrated consistently, the framework should be regarded as a constrained theoretical architecture rather than a settled empirical account.

Finally, the framework does not imply that fragmented or weakly integrated organization is inherently maladaptive, or that distributed relational organization is invariably preferable. Different tasks, environments, developmental histories, and system states may favor different configurations of stabilization and integration. The narrower claim is that enduring memories may differ not only in strength or accessibility, but in the degree and pattern of their incorporation into wider representational structure, and that the accumulation of these differences can shape the architecture within which subsequent learning occurs.

## 5 Discussion

The central contribution of the present article is to shift the analysis of memory formation from trace persistence alone to the organizational fate of eligible information. The framework proposes that enduring mnemonic organization depends both on which eligible activity persists and on the breadth and configuration of its embedding within pre-existing representational structure. Selection and mnemonic architecture are recursively coupled: existing organization biases what becomes eligible, while subsequent stabilization and integration reshape the conditions under which future learning occurs.

This formulation separates memory persistence from memory organization more explicitly than is often done in conventional accounts. Strongly retained, vivid, or readily accessible traces need not be broadly integrated, and their persistence alone does not establish that they will support abstraction, transfer, or flexible relational use. Similar degrees of remembering may therefore contribute to substantially different long-term mnemonic architectures.

The framework does not require selection to be exclusively mnemonic or to be governed by the same influences across developmental stages and system states. Attentional selection, salience detection, and precision weighting may bias which patterns acquire eligibility, while pre-existing mnemonic organization may also constrain what receives learning priority. Neuromodulatory systems may influence several stages of this process and should not be assigned one-to-one to eligibility, selective stabilization, or relational integration (Aston-Jones and Cohen, 2005; Hasselmo, 2006; Friston, 2010). The framework therefore focuses on the architectural consequences of these interacting influences: how they shape what persists and how it is incorporated within an already organized system.

A further contribution lies in distinguishing selective stabilization from relational integration. These dimensions are functionally separable but interacting: selective stabilization concerns the persistence and recurrent availability of eligible activity, whereas relational integration concerns the degree and pattern of its incorporation into wider representational structure. Across repeated learning episodes, their interaction may bias global organization toward more distributed or more fragmented forms, while recurrent engagement within circumscribed domains may produce local-core deepening within either form of organization.

At the empirical level, this architectural distinction suggests contrasts between persistence and integration, local robustness and global incorporation, and accessibility and flexible relational use. The predictions developed above provide an initial basis for testing whether these properties dissociate and whether established architecture systematically influences subsequent learning.

The framework is therefore not proposed as an alternative mechanism to attention, neuromodulation, predictive processing, or established memory systems. It identifies a different explanatory level: how transiently prioritized activity becomes eligible, acquires persistence, and is incorporated in different ways into pre-existing representational structure, and how the accumulation of these outcomes shapes the architecture within which later learning occurs. Neurobiological mechanisms constrain and implement these processes without being isomorphic with them. If future work supports systematic dissociations between selective stabilization and relational integration, the distinction may help refine how enduring mnemonic organization is both theoretically described and empirically investigated.
